# Impact of preoperative inflammatory indices and postoperative pneumonia on postoperative atrial fibrillation in patients with non-small cell lung cancer: a retrospective study

**DOI:** 10.1186/s12890-024-03174-8

**Published:** 2024-07-23

**Authors:** Yingding Ruan, Jianwei Han, Aiming Yang, Qingguo Ding, Ting Zhang

**Affiliations:** 1 Department of Thoracic Surgery, The First People’s Hospital of Jiande, Jiande, China; 2 Radiotherapy Department, Second Affiliated Hospital, Zhejiang University School of Medicine, No. 88 Jiefang Road, Hangzhou, 310009 China

**Keywords:** Non-small cell lung cancer, Pulmonary surgery, Postoperative atrial fibrillation, Preoperative inflammatory indices, Postoperative pneumonia

## Abstract

**Background:**

This study aimed to evaluate the impact of preoperative inflammatory indices and postoperative pneumonia (POP) on postoperative atrial fibrillation (POAF) in non–small cell lung cancer (NSCLC) patients.

**Methods:**

All consecutive patients who underwent pulmonary resection at our hospital (January 2016-October 2019) were enrolled. Preoperative inflammatory indices, demographic data, surgical details, and postoperative conditions were analyzed. Univariate and multivariate analyses of risk factors associated with POAF were also conducted.

**Results:**

Among the 382 patients included in the study, 32 (8.38%) developed POAF. Compared to non-POAF patients, POAF patients had greater incidence of POP (*P* = 0.09). Approximately 31 patients (96.9%) developed atrial fibrillation within three days after surgery. The POAF group had a significantly greater mean age (68.94 years) than did the non-POAF group (63 years) (*P* = 0.002). Additionally, compared to non-POAF patients, POAF patients exhibited an increased number of resected mediastinal lymph nodes (*P* < 0.001) and mediastinal lymph node stations (*P* < 0.001).The POAF group also had a greater intraoperative blood volume (*P* = 0.006), longer surgical duration (*P* = 0.022), and greater drainage volume (*P* = 0.003). IA/B stage (*P* < 0.001) and IIIA/B stage(*P* < 0.001), and lobectomy resection (*P* = 0.008) and wedge resection (*P* = 0.023) were also associated with POAF. Compared to those in the non-POAF group, the POAF group had longer postoperative hospital stays (10.54 days vs. 9 days; *P* = 0.001) and longer drainage times (7 days vs. 5 days; *P* = 0.004). Multivariate analysis revealed age, POP, and stage IIIA/B as independent influencing factors of POAF in NSCLC patients.

**Conclusion:**

Preoperative inflammatory indices were not significantly associated with POAF, but age, POP, and stage IIIA/B were identified as independent influencing factors. Advanced-stage NSCLC patients may have a greater susceptibility to POAF than early-stage patients, although further validation is needed. Additionally, POAF was linked to a longer postoperative hospital stay.

## Introduction

Postoperative atrial fibrillation (POAF) is a common cardiac complication after lung cancer surgery, the reported incidence of which ranges from 2.0–8.6% [1–4]. It can result in life-threatening events such as cardiopulmonary complications, including heart failure, pulmonary embolism, stroke, and fatality, leading to prolonged hospitalization, heightened mortality, and unfavorable prognosis [5]. Therefore, investigating the risk factors for POAF following lung cancer surgery is crucial.

Previous studies have linked the occurrence of POAF after lung surgery to factors such as age, type of lung resection, sex, clinical stage, history of coronary artery disease, and lymph node dissection [1–7]. However, the relationships between POAF development and preoperative and intraoperative risk factors have not yet been firmly established. Moreover, there is limited research on the impact of preoperative inflammatory indices and postoperative pneumonia (POP) on POAF. Therefore, it is important to re-evaluate the risk factors for POAF in recent lung cancer surgeries. The purpose of this study was to characterize the influence of preoperative inflammatory indices, mediastinal lymph node status, and POP on the development of POAF in patients with non-small cell lung cancer (NSCLC).

## Methods

This retrospective cohort study primarily analyzed patients who underwent pulmonary resection at our hospital from January 2016 to October 2019. All pulmonary resections were performed by the same thoracic surgical team throughout the study. The inclusion criteria for patients were: (1) had pulmonary resection and (2) had a postoperative pathological diagnosis of NSCLC. Exclusion criteria included: (1) repeat surgery; (2) benign pathology; (3) non-primary lung tumors; (4) antibiotics or hormone therapy; (5) preoperative radiotherapy, chemotherapy, immunotherapy, targeted therapy, or other treatments; (6) consecutive surgeries within one month; (7) transfer to another hospital; (8) history of atrial fibrillation or pacemaker implantation; (9) stage IV or palliative surgery. Blood samples were collected from all patients within three days before surgery for inflammatory index evaluation.

All patients were restaged according to the eighth edition of the tumor, node, and metastasis (TNM) classification of lung cancer established by the International Association for the Study of Lung Cancer (IASLC) [8].

This study was approved by the Ethics Committee of The First People’s Hospital of Jiande (Ethics Committee Approval Number: 20,230,607,001). Written informed consent was obtained for each participant.

### Data collection

Demographics, clinicopathologic features and operative details of the patients, including sex, age, body mass index(BMI), smoking history, Comorbidities (hypertension, diabetes, coronary heart disease, emphysema, chronic obstructive pulmonary disease), surgical approach, TNM stage, resection sites and type of lung resection, electrocardiogram, 24-hour Holter monitoring, number of mediastinal lymph nodes retrieved and nodal stations explored, surgical duration, intraoperative bleeding volume, drainage time and volume, postoperative hospital stay, POP, pathological types and TNM stages, were retrospectively collected.

The preoperative inflammatory indices evaluated in this study included the neutrophil-to-lymphocyte ratio (NLR), platelet-to-lymphocyte ratio (PLR), lymphocyte-to-monocyte ratio (LMR), and systemic immune inflammation index (SII), calculated as follows: SII = platelet * neutrophil/lymphocyte. These indices were used to assess the patients’ preoperative systemic inflammatory status.

### Electrocardiographic (ECG) monitoring

All patients underwent ECG monitoring for 24 to 72 h after surgery. If arrhythmias were detected or if patients experienced symptoms such as palpitations or chest tightness, bedside electrocardiography was performed to confirm the diagnosis.

### Definition of POAF

POAF was defined as the occurrence of new-onset atrial fibrillation confirmed by an ECG at least once between the thoracic procedure and discharge from the hospital [9]. The diagnosis of POAF was based on an ECG assessment.

### Definition of POP

Patients with postoperative pneumonia were defined as those who did not present with preoperative pneumonia and exhibited new infiltrations on the postoperative computed tomography images. At least three of the following criteria were required to confirm the diagnosis of POP: (1) Chest plain film or chest computed tomography (CT) showing lung exudation and consolidation; (2) Temperature > 38 °C; (3) White blood cells(WBC) > 10,000/mm^3^ or < 3000/mm^3^; (4) Pathogens were detected in sputum, or purulent secretions were detected via bronchoscopy [9].

### Statistical analysis

The data are presented as the mean and standard deviation or median (P25, P75). Student’s t test was used for normally distributed data, while the Wilcoxon rank-sum test was used for abnormally distributed data. The frequency (%) was calculated for categorical data and was analyzed using the chi-square test or Fisher’s exact probability method. Univariate and multivariate analyses were performed using the binary logistic regression model. Variables with *P* < 0.1 in the single factor analysis were included in the multiple factor analysis, and *P* < 0.05 was considered to indicate statistical significance. IBM SPSS Statistics Version 26 software (SPSS, Chicago, IL, USA) was used for all the data analyses.

## Results

### Demographic and baseline characteristics

**Fig. 1:**
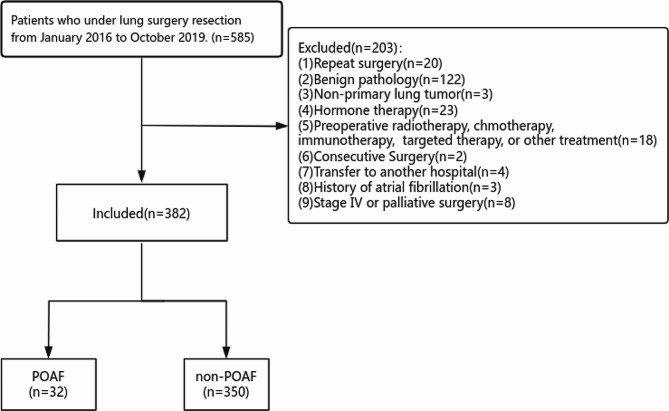
Flow diagram showing the schema of study selection for patients with NSCLC (POAF, postoperative atrial fibrillation; NSCLC, non-small cell lung cancer )

Between January 2016 and October 2019, approximately 585 pulmonary resections were performed at our hospital. After excluding 203 patients, 382 patients were included in the analysis (Fig. 1). A total of 199 females and 183 males were included. The median age was 65 years (range: 57.35–70.57), and the median BMI was 22.7 kg/m² (range: 20.9–24.9). The average preoperative inflammatory markers were as follows: pSII, 431.76 (range: 292.73-623.18); PLR, 128.71 (range: 97.27-172.78); NLR, 2.57 (range: 1.84–3.46); and LMR, 4 (range: 3-5.21). The median surgical duration was 138 min (range: 99–180), and the average intraoperative bleeding volume was 100 ml (range: 50–100). Among the included patients, 316 had adenocarcinoma, 47 had squamous cell carcinoma, and 19 had other rare NSCLC subtypes. Postoperative TNM stages were as follows: IA/B(88.3%), IIA/B (5.2%) and IIIA/B (6.5%). Pulmonary resections included pneumonectomy (1.3%), wedge resection (17.8%), segmental resection (18.1%), and lobectomy (62.8%). The median mediastinal lymph node and node station counts were 5 (range: 0–10) and 2 (range: 0–3), respectively. The median drainage time and postoperative hospital stay were 5 days (range: 3.2-9) and 9.4 days (range: 6.4–12.4), respectively. POP was the most common postoperative complication and affected approximately 66 patients. The detailed data are available in Table 1.

**Table 1:** Patient characteristics, incidence of POAF, [n(%),M(P25,P75)]

Characteristics	Data
Incidence of POAF, n(%)	32(8.4)
Time of incidence(days)	1(1,3)
Sex, n(%)	
Male	183(47.5)
Female	199(52.5)
Age(years)	65.00(57.35, 70.57)
BMI(kg/m^2^)	22.70(20.30, 24.90)
Postoperative hospital stay(days)	9.4(6.4, 12.4)
Smoking, n(%)	122(31.9)
Arrhythmia, n(%)	54(14.1)
Comorbidities, n(%)	
Hypertension	92(24.1)
Diabetes	6(1.6)
Coronary heart disease	4(1.0)
Chronic Obstructive Pulmonary Disease	21(5.5)
Comorbidities of Hypertension, Diabetes, Cardiac History, and Pulmonary History	35(9.2)
TNM Stage, n(%)	
Stage IA/B	337(88.3)
Stage IIA/B	20(5.2)
Stage IIIA/B	25(6.5)
Approaches, n(%)	
U-VATS	212(55.5)
M-VATS	159(41.6)
Thoracotomy	11(2.9)
Type of lung resection, n(%)	
Lobectomy	240(62.8)
Segmental	69(18.1)
Wedge	68(17.8)
Pneumonectomy	5(1.3)
Resection Site, n(%)	
Right upper	122(31.9)
Right middle	26(6.8)
Right lower	76(19.9)
Left upper	91(23.8)
Left lower	62(16.2)
Pneumonectomy, n(%)	5(1.3)
Duration of surgery (min)	138.00(99.21, 180.42)
Intraoperative bleeding volume (mL)	100.00(50.00, 100.00)
Pathological Types, n(%)	
Adenocarcinoma	316(82.7)
Squamous Cell Carcinoma	47(12.3)
Rare NSCLC	19(5.0)
Number of mediastinal lymph nodes retrieved	5(0, 10)
Mediastinal lymph node stations explored	2(0, 3)
Postoperative Complications, n(%)	119(31.2)
Drainage volume (mL)	860.00(527.51, 1417.53)
Drainage time (days)	5.00(3.21,9.00)
Antibiotics(%)	338(88.5)
pSII	431.76(292.73,623.18)
PLR	128.71(97.27,172.78)
NLR	2.57(1.84,3.46)
LMR	4.00(3.00,5.21)
Preoperative albumin level(g/L)	43.15(39.85,46.55)
Postoperative albumin level(g/L)	33.80(30.61, 36.65)
Echocardiography ejection fraction(%)	67.72(63.38, 73.00)
Echocardiography left atrial size(mm)	31.51(28.73, 35.70)
Postoperative Pneumonia(%)	66(17.3)

BMI, Body Mass Index; POAF, postoperative arterial fibrillation; NSCLC, non-small cell lung cancer; TNM, Tumor, Node, and Metastasis; U-VATS, Uniportal Video-assisted Thoracoscopic Surgery; M-VATS, Multiportal Video-assisted Thoracoscopic Surgery; pSII, Preoperative Systemic Immune Inflammation index NLR, Neutrophil-to-Lymphocyte Ratio; PLR, Platelet-to-Lymphocyte Ratio; LMR, Lymphocyte-to-Monocyte Ratio; Arrhythmia, Arrhythmias excluding Atrial Fibrillation; M(P25,P75), Median(25th percentile,75th percentile)

### POAF outcome

Among the 382 NSCLC patients, 32 (8.4%) developed POAF. No in-hospital POAF-related deaths occurred. POAF symptoms developed within a median of 1 day (range: 1–3) post-surgery and lasted approximately 1 day on average. Among these patients, 3 reported palpitations and chest discomfort, while the others were asymptomatic. Blood flow dynamics were normal across all patients. Among the 26 patients treated with amiodarone or digoxin, clinical improvement was observed in all patients. Of the 32 patients, 29 had a restored sinus rhythm before discharge, At discharge, 3 patients still had POAF and were prescribed anticoagulants.

### Comparison of clinical data between the POAF and non-POAF groups

The study revealed differences in factors between the POAF and non-POAF groups. The average age was greater in the POAF group than in the non-POAF group (68.94 ± 7.808 years vs. 63.00 ± 10.435 years, *P* = 0.002). More mediastinal lymph nodes and stations were retrieved in the POAF group [median nodes: 9.5 (5.5, 14) vs. 4 (0, 9), *P* < 0.001; average node stations: (3.28 ± 2.004) vs. (1.94 ± 1.766), *P* < 0.001]. Furthermore, the duration of surgery (*P* = 0.022), intraoperative bleeding volume (*P* = 0.006), postoperative hospital stay (*P* = 0.001), drainage volume (*P* = 0.003), and drainage time (*P* = 0.004) were significantly greater in patients with POAF than in patients without POAF. Additionally, TNM stage (*P* < 0.001) and the type of lung resection (*P* = 0.049) differed between the two groups.

However, there were no differences in preoperative inflammatory factor levels, such as the pSII, PLR, NLR, or LMR, between the two groups. Similarly, comorbidities such as hypertension, diabetes, coronary heart disease, chronic obstructive pulmonary disease, and emphysema had minimal impacts on POAF, and these two conditions were not significantly different between the groups. Smoking status and BMI were also not significantly different (*P* > 0.05). Other factors, including sex, pathological type, surgical approach, left atrial size, ejection fraction, antibiotics, and preoperative and postoperative albumin (ALB) concentrations, also did not exhibit substantial variation (*P* > 0.05). The detailed results are available in Table 2.

**Table 2:** Patient characteristics and statistical analysis[n(%),mean ± standard deviation, M(P25,P75)]

Variables	POAF(*n* = 32)	non-POAF(*n* = 350)	t/Z	*P* Value
Sex, n(%)				0.537
Male	17(53.1)	166(47.4)		
Female	15(46.9)	184(52.6)		
Age(years)	68.94 ± 7.808	63.00 ± 10.435	3.138	0.002
BMI(kg/m^2^)	22.23 ± 3.867	22.79 ± 3.571	-0.849	0.397
Smoking, n(%)				0.271
Yes	13(40.6)	109(31.3)		
No	19(59.4)	241(68.9)		
Arrhythmia, n(%)				0.434
Yes	6(18.8)	48(13.7)		
No	26(81.2)	302(86.3%)		
TNM Stage, n(%)				< 0.001
Stage IA/B	22(68.8)	315(90.0)		< 0.001
Stage IIA/IIB	2(6.2)	18(5.1)		0.788
Stage IIIA/IIIB	8(25.0)	17(4.9)		< 0.001
Pathological types, n(%)				0.755
Adenocarcinoma	26(81.3)	290(82.9)		0.818
Squamous cell carcinoma	5(15.6)	42(12.0)		0.550
Rare NSCLC	1(3.1)	18(5.1)		0.615
Resection Site, n(%)				0.648
Right upper	11(34.4)	111(31.7)		0.757
Right middle	4(12.5)	22(6.3)		0.181
Right lower	4(12.5)	72(20.6)		0.274
Left upper	7(21.9)	84(24.0)		0.787
Left lower	6(18.8)	56(16.0)		0.686
Type of lung resection, n(%)				0.049
Lobectomy	27(84.4)	213(60.9)		0.008
Segmental	4(12.5)	65(18.6)		0.392
Wedge	1(3.1)	67(19.1)		0.023
Pneumonectomy	0(0.0)	5(1.4)		0.496
Approach, n(%)				0.144
U-VATS	13(40.6)	199(56.9)		0.451
M-VATS	17(53.1)	142(40.6)		0.168
Thoracotomy	2(6.3)	9(2.5)		0.234
Comorbidities, n(%)				0.774
Yes	14(43.8)	144(41.1)		
No	18(56.3)	206(59.9)		
Antibiotics, n(%)				0.447
Yes	27(84.4)	311(88.9)		
No	5(15.6)	39(11.1)		
Postoperative Pneumonia, n(%)				0.090
Yes	9(28.1)	57(16.3)		
No	23(71.9)	293(83.7)		
Duration of surgery(mL)	166.22 ± 61.458	141.23 ± 58.363	2.309	0.022
Mediastinal lymph node stations explored	3.28 ± 2.004	1.94 ± 1.766	4.073	< 0.001
Preoperative albumin level(g/L)	41.73 ± 6.148	43.15 ± 4.488	-1.647	0.213
Postoperative albumin level(g/L)	32.80 ± 3.664	33.76 ± 4.309	-1.223	0.222
Echocardiography ejection fraction(%)	67.13 ± 8.749	68.48 ± 7.134	-0.999	0.318
Echocardiography left atrial size(mm)	32.98 ± 4.651	31.85 ± 5.519	1.125	0.261
Intraoperative bleeding volume (mL)	100.00(100.00, 200.00)	100.00(50.00, 100.00)	-2.745	0.006
Postoperative hospital stay(days)	10.54(8.44, 15.36)	9.00(5.91, 12.40)	-3.293	0.001
Number of mediastinal lymph nodes retrieved	9.50(5.50, 14.00)	4.00(0.00, 9.00)	-3.873	< 0.001
Drainage volume (mL)	1150.00(791.25, 2192.50)	820.00(543.75, 1400.00)	-2.977	0.003
Drainage time (days)	7.00(5.00, 10.75)	5.00(3.03, 9.00)	-2.865	0.004
pSII	368.24(225.37, 599.49)	436.15(303.05, 624.03)	-1.507	0.132
PLR	118.86(92.57, 158.21)	129.21(97.27, 174.72)	-0.835	0.404
LMR	4.42 ± 1.906	4.26 ± 1.778	0.489	0.625
NLR	2.47(1.75, 3.31)	2.57(1.86, 3.47)	-0.553	0.580

BMI, Body Mass Index; U-VATS, Uniportal Video-assisted Thoracoscopic Surgery; M-VATS, Multiportal Video-assisted Thoracoscopic Surgery; pSII, Preoperative Systemic Immune Inflammation index; NLR, Neutrophil-to-Lymphocyte Ratio; PLR, Platelet-to-Lymphocyte Ratio; LMR, Lymphocyte-to-Monocyte Ratio; M(P25,P75), Median(25th percentile,75th percentile)

### Risk factor analysis for POAF

We conducted univariate and multivariate logistic regression analyses of the clinical data. Univariate analysis revealed significant differences (*P* < 0.1) in age, TNM stage, mediastinal lymph node stations and nodes, surgery duration, drainage volume and time, postoperative hospitalization, type of lung resection, and POP. Importantly, multivariate logistic regression analysis(*P* < 0.05) revealed that age (Exponential(B)(Exp(B) = 1.08, 95% confidence interval(CI) = 1.02–1.14, *P* = 0.009), stage IIIA/B (Exp(B) = 5.81, 95% CI = 1.68–20.10, *P* = 0.005), and POP (Exp(B) = 2.63, 95% CI = 1.04–6.65, *P* = 0.039) were found to be independent influencing factors of POAF in patients with NSCLC (Tables 3 and 4).

**Table 3:** Results of univariate logistic regression analyses for POAF

Variables	*P* value	Exp(B)	95%Exp(B) CI	
			Down	Up
Sex				
Male	1			
Female	0.538	0.80	0.39	1.64
Age(years)	0.002	1.07	1.03	1.12
Pathological Types				
Adenocarcinoma	1			
Squamous cell carcinoma	0.582	1.33	0.48	3.65
Rare NSCLC	0.648	0.62	0.08	4.83
Arrhythmia				
No	1			
Yes	0.436	1.45	0.57	3.71
TNM Stage				
Stage IA/IB	1			
Stage IIA/IIB	0.550	1.59	0.35	7.30
Stage IIIA/IIIB	< 0.001	6.74	2.62	17.33
BMI(kg/m^2^)	0.395	0.96	0.86	1.06
Intraoperative bleeding volume(mL)	0.020	1.01	1.01	1.01
Duration of surgery(min)	0.023	1.01	1.01	1.01
Postoperative hospital stay(days)	< 0.001	1.10	1.04	1.16
Resection Site	0.660	0.95	0.75	1.20
Right upper	1			
Right middle	0.334	0.55	0.16	1.87
Right lower	0.337	1.78	0.55	5.82
Left upper	0.731	1.19	0.44	3.20
Left lower	0.884	0.92	0.33	2.63
Type of lung resection				
Lobectomy	1			
Segmental	0.192	0.49	0.16	1.44
Wedge	0.037	0.12	0.02	0.88
Pneumonectomy	0.989	0.00	0.00	
Approach				
U-VATS	1			
M-VATS	0.115	1.83	0.86	3.89
Thoracotomy	0.141	3.40	0.67	17.39
Comorbidities				
No	1			
Yes	0.770	0.90	0.43	1.87
Number of mediastinal lymph nodes retrieved	< 0.001	1.09	1.04	1.14
Mediastinal lymph node stations explored	< 0.001	1.46	1.20	1.77
Postoperative Pneumonia				
No	1			
Yes	0.095	0.50	0.22	1.13
Drainage volume(mL)	0.007	1.01	1.01	1.01
Drainage time(days)	0.028	1.07	1.01	1.14
pSII	0.451	1.00	1.00	1.00
PLR	0.483	1.00	0.99	1.00
NLR	0.384	0.90	0.71	1.14
LMR	0.624	1.05	0.86	1.28
Preoperative albumin level(g/L)	0.102	0.94	0.87	1.01
Postoperative albumin level(g/L)	0.222	0.95	0.87	1.03
Smoking				
No	1			
Yes	0.273	1.51	0.72	3.17
Antibiotics				
No	1			
Yes	0.450	1.48	0.54	4.06
Echocardiography ejection fraction(%)	0.317	0.97	0.93	1.03
Echocardiography left atrial size(mm)	0.261	1.04	0.97	1.11

POAF, postoperative arterial fibrillation; BMI, Body Mass Index; U-VATS, Uniportal Video-assisted Thoracoscopic Surgery; M-VATS, Multiportal Video-assisted Thoracoscopic Surgery; pSII, Preoperative Systemic Immune Inflammation index; NLR, Neutrophil-to-Lymphocyte Ratio; PLR, Platelet-to-Lymphocyte Ratio; LMR, Lymphocyte-to-Monocyte Ratio; Exp(B), Exponential(B); CI, Confidence Interval

**Table 4:** Results of multivariable logistic regression analyses for POAF

Variables	*P* value	Exp(B)	95%Exp(B) CI	
			Down	Up
Age(years)	0.009	1.08	1.02	1.14
TNM Stage				
Stage IA/B	1			
Stage IIA/IIB	0.783	0.77	0.15	4.24
Stage IIIA/IIIB	0.005	5.81	1.68	20.10
Intraoperative bleeding volume(mL)	0.455	1.00	1.00	1.00
Duration of surgery(min)	0.589	1.00	0.99	1.01
Type of lung resection				
Lobectomy	1			
Segmental	0.512	1.56	0.41	5.94
Wedge	0.438	0.42	0.05	3.81
Pneumonectomy	0.988	0.00	0.00	
Number of Mediastinal lymph nodes retrieved	0.067	1.07	1.00	1.14
Mediastinal lymph node stations explored	0.554	1.09	0.81	1.48
Postoperative Pneumonia				
No	1			
Yes	0.039	2.63	1.04	6.65
Drainage volume(mL)	0.772	1.00	1.00	1.00
Drainage time(days)	0.420	0.95	0.82	1.08

POAF, postoperative arterial fibrillation; U-VATS, Uniportal Video-assisted Thoracoscopic Surgery; M-VATS, Multiportal Video-assisted Thoracoscopic Surgery; Exp(B), Exponential(B); CI, Confidence Interval

## Discussion

Despite ongoing efforts to reduce POAF, it remains a significant complication after pulmonary resection. Our study revealed an 8.4% incidence of POAF in NSCLC, supporting the prevalence of POAF [1–4]. While research on POAF has decreased in recent years, this topic remains important. By analyzing preoperative inflammatory markers, POP incidence, and other risk factors, we found that POP, age, and stage IIIA/B were significant factors affecting POAF. These findings validate the findings of previous research and provide guidance for prevention and treatment.

Previous studies have suggested that local inflammation is a risk factor for POAF [10–12]. Earlier research has linked the occurrence of POAF to interleukin-2, interleukin-6, and C-reactive protein [12–14]. Boons et al. [11]. reported associations between prolonged ventilation and pneumonia combined with POAF in cardiothoracic surgery patients. Preventive measures such as low-flow oxygen inhalation may reduce the incidence of POAF, although this remains controversial [15]. However, no studies investigating the association between POP and POAF were found in the literature search. In our study, the incidence of POP was 17.3%, which falls within the reported range of 1.7–24.3% in previous studies [16–19]. In addition, in our study, POP accounted for the highest proportion among all postoperative complications (55.5%) (66/119). Moreover, a greater proportion of POP was observed in the POAF group than in the non-POAF group (28.1% vs. 16.3%, *p* = 0.09), indicating a correlation between POP and POAF occurrence. Through multivariate analysis, we obtained preliminary evidence that POP is indeed an independent risk factor for POAF (*P* = 0.039).

On the other hand, preoperative inflammatory markers, such as white blood cell count, are widely recognized as predictive factors for POAF [20, 21]. However, related research on the impact of preoperative inflammatory indices on the development of POAF following lung cancer surgery is rare. In this study, the main preoperative inflammatory indices used were the pSII, PLR, NLR, and LMR. To some extent, they represent the preoperative inflammatory level in patients. Additionally, there is evidence that these indices can predict the occurrence of POP [9]. Although this study revealed no impact of inflammatory indices on POAF, further research is needed to determine whether these indices can predict the occurrence of POP, which in turn may affect the development of POAF.

Age has consistently proven to be the most significant factor influencing the incidence of POAF. In our study, the average age of the POAF group (68 years) was significantly higher than that of the non-POAF group (63 years), which aligns with previous research showing a positive correlation between age over 60 and the incidence of POAF among chest surgery patients [22].Meanwhile, multivariate analysis in our study showed that the incidence of POAF was positively correlated with age (Exp(B) = 1.08), which is consistent with the results of previous studies [1, 5, 7]. However, there are various confounding factors intertwined in the multivariate analysis, emphasizing the necessity for further exploration of the relationship between age and POAF, especially considering other potential influencing factors and their interactions.

In 2014, a study conducted by Jelena Ivanovic et al. [23]. revealed that out of 274 patients, 43 had atrial fibrillation. Based on the American Joint Committee on Cancer(AJCC) 7th edition 2009 staging system, patients were categorized into stages IA/IB, IIA/IIB, IIIA/IIIB, and IV. The results revealed a statistically significant difference between patients with and non- POAF only in stage IA/IB (*P* = 0.05) and stage IV (*P* < 0.05). In 2010, Onaitis et al. [24]. conducted a study utilizing the Thoracic Surgeons General Thoracic Surgery Database, focusing on patients with lung cancer who underwent lobectomy or pneumonectomy. Patients were categorized based on TNM staging into those with pathological stages less than stage II and those with pathological stages greater than stage II. Multivariate analysis indicated that patients with higher-stage or larger tumors were more prone to developing POAF. Thus, TNM staging is indeed related to POAF. In our study, we also found a relationship between TNM stage and POAF (*P* < 0.001). Multivariate analysis revealed that stage IIIA/B is an independent risk factor for POAF. However, since we excluded Stage IV patients, further prospective, multicenter studies with a larger sample size are needed to investigate whether stage IV exacerbates the impact on POAF.

Research has suggested that the volume of the resected lung may affect POAF. One study [3] revealed that the incidence of POAF was lower in patients who underwent segmental resection (1.4%) than in those who underwent lobectomy resection (2.8%). Furthermore, some studies [2, 25] identified an increasing extent of lung resection as an independent risk factor for POAF. Several studies have proposed a potential association between tissue loss during pulmonary resection, postoperative reduction in lung function, myocardial hypoxia, and POAF [4, 7]. In our study, the results revealed a statistically significant difference between the POAF and non-POAF groups (*P* = 0.049). However, we observed only one patient with wedge resection in the POAF group. Therefore, after excluding wedge resection patients, the analysis revealed no significant difference between the two groups (*p* = 0.309). Regardless of the aforementioned circumstances, multivariate analysis revealed that the type of lung resection was not an independent risk factor for POAF. Interestingly, no patients in the POAF group underwent pneumonectomy. Only 5 (1.3%) of the included patients underwent left-sided pneumonectomy. However, pneumonectomy has been reported as a risk factor for POAF [23]. Therefore, the type of lung resection may impact the occurrence of POAF, but the limited number of patients who underwent pneumonectomy and wedge resection restricts this comparison.

Mediastinal lymph node dissection may cause direct damage to the cardiac nerve plexus at the aortic arch and tracheal bifurcation, thereby affecting the incidence of POAF. Some experts [1, 2, 26] have hypothesized that mediastinal lymph node dissection could trigger atrial fibrillation, increasing the risk of POAF. However, a study [27] argued that there is no significant association between them.

We separately analyzed the number of mediastinal lymph nodes and the number of lymph node stations explored. The POAF group had a significantly greater median number of mediastinal lymph nodes (9 vs. 4) and average number of detected lymph node stations (3 vs. 2) than did the non-POAF group (*P* < 0.05). However, multivariate analysis demonstrated that the impact of the number of lymph nodes and the number of explored lymph node stations on POAF was minimal. Therefore, further research is still needed to investigate whether omitting mediastinal lymph node dissection reduces the occurrence of POAF.

Recent studies have indicated that solid tumors measuring 2 cm or larger require lobectomy with mediastinal lymph node dissection or sampling, while for ground-glass nodules smaller than 2 cm, wedge resection or segmentectomy is feasible with no need for mediastinal lymph node evaluation [24, 28, 29]. Consequently, for patients with larger tumors and more advanced TNM staging, the extent of thoracic trauma increases, potentially influencing the occurrence of POAF.

Both the type of lung resection and mediastinal lymph node dissection can influence the duration of surgery. Previous research has indicated an increased incidence of POAF when the surgical duration exceeds 180 min [19, 21]. In our study, the average surgical durations for the POAF and non-POAF groups were approximately 166 min and 141 min, respectively. Although there was a statistically significant difference between the two groups (*P* = 0.022), multivariate analysis showed that it had no impact on POAF. This finding is consistent with the aforementioned research since the average surgical duration in both groups did not exceed 180 min. Therefore, we still recommend attempting to maintain the surgical duration within 180 min whenever possible.

Previous studies have consistently reported a correlation between POAF and prolonged postoperative hospital stay [2]. Our research revealed that patients who developed POAF had a median postoperative hospital stay of approximately 11 days, whereas patients who did not develop POAF had a median hospital stay of 9 days (*P* = 0.001). Additionally, the drainage time in the POAF group was significantly prolonged, and the drainage volume was greater. Univariate analysis also demonstrated correlations between postoperative hospital stay (*P* = 0.001), drainage time (*P* < 0.001), drainage volume (*P* < 0.001), and POAF. Although multivariate analysis did not reveal drainage time or volume to be independent risk factors for POAF, prolonged drainage time and increased drainage volume during hospitalization can lead to a longer hospital stay, which can also have an impact on POAF incidence. Therefore, further multicenter data collection and prospective studies are necessary.

Previous studies have consistently indicated that POAF often occurs within three days after surgery [1, 2]. Our research revealed that the incidence of POAF was highest on the first day after surgery (65.6%), with 31 out of 32 cases occurring within three days after surgery. This early occurrence may be exacerbated by increased sputum production within 24–72 h after surgery, potentially leading to acute hypoxemia. Pain and anesthesia directly affect respiratory and cough reflexes, increasing the incidence of POAF. Notably, POAF typically diminishes over time and is usually transient after pulmonary resection surgery. It is often caused by surgical trauma, hypoxemia, or other stressors rather than organic heart disease. Therefore, POAF following pulmonary resection surgery is typically transient.

Amiodarone and digoxin have been recommended by researchers for the effective management of POAF [10]. Our study assessed the efficacy of these agents in the treatment of POAF. Among the 26 patients treated with amiodarone or digoxin, clinical improvement was observed in all patients. Of the 32 patients, 29 had a restored sinus rhythm before discharge, while 3 continued to have atrial fibrillation and received anticoagulant therapy after discharge. Some patients reported symptoms of palpitations and chest discomfort, but all patients maintained hemodynamic stability and did not experience significant discomfort.

This study has several strengths. Firstly, there is limited research on preoperative inflammatory indices, and this study innovatively incorporated preoperative inflammatory indices into the analysis. Although the results showed no correlation with POAF, the inclusion of preoperative inflammatory indices opens up a new avenue for future research as our understanding of inflammatory indices deepens. Secondly, we specifically focused on the impact of POP on POAF. Although our results indicated that POP has an influence on POAF, further validation is required through prospective, multicenter studies.

This study has several limitations. Firstly, due to the retrospective enrollment of patients from a single center, there is a possibility of selection bias, potentially resulting in the exclusion or underdetection of patients with POAF. Secondly, incomplete data were collected for outcomes such as lung function and pain, limiting their examination. Thirdly, the sample size was small, and the incidence of POAF was limited, which affected the comprehensive analysis of risk factors. Lastly, for the indicators of antibiotics, we did not conduct further detailed analysis, such as the type, dosage, or duration of treatment. Therefore, in our future work, we will delve into these aspects to gain a better understanding of their impact on postoperative recovery.

In summary, the results of this study demonstrated no significant association between preoperative inflammatory indices and the POAF. However, stage IIIA/B, age, and POP were identified as independent influencing factors of POAF. Preliminary findings advanced-stage NSCLC patients may have a greater susceptibility to POAF than early-stage patients, but further validation is needed. Additionally, POAF was found to be associated with prolonged hospital stays.

## Data Availability

Any researchers interested in this study could contact Yingding Ruan (E-mail: ruanyingding@sina.com) to request the data.

## Declarations

### AI and AI-assisted technologies

In the preparation of this work, the authors did not utilize any AI technology for editing the manuscript. The authors take full responsibility for the content of the publication.

## Abbreviations

POAF: Postoperative Arterial Fibrillation
NSCLC: Non-Small Cell Lung Cancer
BMI: Body Mass Index
POP: Postoperative Pneumonia
pSII: Preoperative Systemic Immune Inflammation Index
NLR: Neutrophil-to-Lymphocyte Ratio
PLR: Platelet-to-Lymphocyte Ratio
LMR: Lymphocyte-to-Monocyte Ratio
TNM: Tumor, Node, and Metastasis
IASLC: International Association for the Study of Lung Cancer
ECG: Electrocardiography
ALB: Albumin
